# Body Mass Index (BMI) Is the Strongest Predictor of Systemic Hypertension and Cardiac Mass in a Cohort of Children

**DOI:** 10.3390/nu15245079

**Published:** 2023-12-12

**Authors:** Marianna Fabi, Matteo Meli, Davide Leardini, Laura Andreozzi, Giulio Maltoni, Maria Bitelli, Luca Pierantoni, Chiara Zarbo, Arianna Dondi, Cristina Bertulli, Luca Bernardini, Andrea Pession, Marcello Lanari

**Affiliations:** 1 Pediatric Emergency Unit, IRCCS Azienda Ospedaliero—Universitaria di Bologna, 40138 Bologna, Italy; marianna.fabi@aosp.bo.it (M.F.); laura.andreozzi4@unibo.it (L.A.); luca.pierantoni@aosp.bo.it (L.P.); arianna.dondi@aosp.bo.it (A.D.); marcello.lanari@unibo.it (M.L.); 2 Specialty School of Paediatrics, Alma Mater Studiorum, University of Bologna, 40138 Bologna, Italy; maria.bitelli@studio.unibo.it (M.B.); chiarazarbo@gmail.com (C.Z.); luca.bernardini10@studio.unibo.it (L.B.); 3 Pediatric Hematology and Oncology, IRCCS Azienda Ospedaliero—Universitaria di Bologna, 40138 Bologna, Italy; davide.leardini3@studio.unibo.it; 4 Department of Medical and Surgical Sciences (DIMEC), University of Bologna, 40138 Bologna, Italy; 5 Pediatric Unit, IRCCS Azienda Ospedaliero—Universitaria di Bologna, 40138 Bologna, Italy; cristina.bertulli@gmail.com (C.B.); andrea.pession@unibo.it (A.P.)

**Keywords:** systemic hypertension, children, risk factors, overweight, obesity, left ventricle mass

## Abstract

Background: Hypertension (HTN) is a well-established cardiovascular (CV) risk factor in adults. The presence of HTN in children appears to predict its persistence into adulthood. Early diagnosis of HTN is crucial to reduce CV morbidity before the onset of organ damage. Aim: The aim of this study is to investigate cardiac damage in HTN, its risk factors (RFs), and evolution. Methods: We conducted a prospective/retrospective study involving children referred to the Childhood Hypertension Outpatient Clinic. This study included clinical and echocardiographic assessments of cardiac morphology and function at three time points: enrollment (T0) and follow-up (T1 and T2). Results: Ninety-two patients (mean age 11.4 ± 3 years) were enrolled. Cardiac eccentric and concentric hypertrophy were present in 17.9% and 9%, respectively, with remodeling in 10.5%. Overweight/obese subjects exhibited significantly higher systolic blood pressure (SBP), frequency of HTN, and body mass index (BMI) at T0 compared with patients with chronic kidney disease (CKD). SBP and BMI persisted more during follow-up. Normal-weight vs. overweight/obese patients were significantly more likely to have normal geometry. Positive correlations were found between BMI and left ventricular (LV) mass at T0, BMI and SBP at T0 and T1. Gender, BMI, SBP, and diastolic blood pressure (DBP) significantly predicted LV mass index (LVMI), but only BMI added significance to the prediction. During follow-up, the variation of BMI positively correlated with the variation of SBP, but not with LVMI. Conclusions: In our cohort, body weight is strongly associated with HTN and cardiac mass. Importantly, the variation in body weight has a more significant impact on the consensual variation of cardiac mass than blood pressure (BP) values. A strict intervention on weight control through diet and a healthy lifestyle from early ages might reduce the burden of CV morbidity in later years.

## 1. Introduction

Systemic hypertension (HTN) stands as a well-established cardiovascular (CV) risk factor in adults, contributing significantly to morbidity and mortality associated with myocardial infarction, stroke, congestive heart failure, peripheral vascular disease, retinopathy, and end-stage renal disease. Hemodynamic and non-hemodynamic factors play pivotal roles in influencing cardiac function and morphology, often resulting in increased left ventricular (LV) diameters and wall dimensions [1]. Consequently, left ventricular hypertrophy (LVH) and remodeling are prevalent in individuals with HTN. LVH, an independent risk factor for CV events in adults, has also shown a correlation with hypertension in childhood [2,3,4,5,6,7]. In older individuals, increased blood pressure (BP) is commonly attributed to arterial wall stiffening, while in younger individuals, it appears linked to heightened heart rate and left ventricular ejection velocities [8]. Vascular involvement intensifies with the severity of BP categories, impacting both systolic and diastolic function in the youth [9]. Notably, the tracking of BP commences early in life, with hypertension in children being a predictor of its persistence into adulthood [10,11]. In recent decades, the escalating epidemic of overweight and obesity among children and adolescents [12] aligns with a rising prevalence of pre-hypertension and HTN within the same age range, accompanied by an overall increase in BP values [13,14]. Even within the normal range of values, an elevated body mass index (BMI) in late adolescence has been associated with increased vascular mortality in later years [15]. Meta-analysis of data from four large longitudinal cohorts—Bogalusa Heart Study, Muscatine Study, Cardiovascular Risk in Young Finns Study, and Childhood Determinants of Adult Health study—clearly demonstrates the substantial impact of childhood adiposity on CV risk in later life [16]. Moreover, youth with high-normal BP exhibit an increased risk of cardiac and vascular sequelae compared to normotensive counterparts [17]. Several factors, including personal history, perinatal conditions (preterm birth, intra-uterine growth restriction, perinatal complications, and low birth weight), and a family history of CV issues, are associated with HTN in childhood. While personal and perinatal factors remain unmodifiable, weight stands out as a modifiable risk factor through multi-component management aimed at reducing BMI in the overweight/obese pediatric population [18,19,20]. Incorporating physical activity, a balanced diet with a reduced glycemic load, and mindful eating are crucial in addressing obesity. Dietary plans such as the “Dietary Approaches to Stop Hypertension (DASH) Child 1 version” and the use of nutrition-related apps or digital aids can further contribute to weight control [21]. In the pursuit of mitigating HTN, its cardiovascular consequences, and the associated economic burden, early diagnosis is paramount. This study seeks to investigate cardiac damage in HTN, exploring its correlations with known risk factors (RFs), and examining its longitudinal evolution.

## 2. Materials and Methods

This was a monocentric prospective/retrospective study that included all children under 18 years old being monitored for hypertension (HTN) by a multidisciplinary team consisting of a Pediatric Cardiologist, Endocrinologist, and Nephrologist at the Pediatric Department, Scientific Institute for Research and Healthcare (IRCCS) Policlinico di Sant’Orsola, Bologna, Italy. Demographic and anthropometric parameters (age, sex, ethnicity, height, weight, BMI), comorbidities (chronic kidney disease (CKD), endocrine disorders (ED), Obstructive Sleep Apnea Syndrome (OSAS/snoring), personal factors (gestational age, birth weight, intra-uterine growth restriction, twins, delivery complications, perinatal morbidity, cardiovascular and urologic diseases), and family risk for cardiovascular diseases (CVD) were recorded. Overweight was defined based on BMI >85th percentile for age and sex, and obesity when >97th percentile for age and sex [22,23]. For each patient, systolic (SBP) and diastolic arterial blood pressure (DBP) were measured using the auscultatory method and expressed as absolute values and percentiles according to the 2016 European Society of Hypertension [13]. HTN was defined when SBP and/or DBP were at least the 95th percentile for sex, age, and height on at least three separate occasions. High-normal BP was defined when the average SBP and/or DBP was at least the 90th but less than the 95th percentile for children younger than 16 years old. For boys and girls aged 16 or older, HTN was defined for BP values equal to or greater than 140/90 mmHg, and high-normal BP for values in the range 130–139/85–89 mmHg but less than 140/90 mmHg. All subjects underwent electrocardiogram (ECG) and transthoracic echocardiography. Echocardiography, performed by an expert pediatric cardiologist, using a Philips IE33 ultrasound machine with S5-1 Phased Array Ultrasound Probe, adhered to the recommendations of the American Society of Echocardiography and European Association of Cardiovascular Imaging [24,25]. Measurements were conducted over three consecutive cardiac cycles. Parameters including left chambers dimensions (interventricular septal width at end-diastole, IVSD; left ventricle end-diastole diameter, LVEDD; left ventricle end-systole diameter, LVESD; posterior wall thickness at end-diastole, PWD; left atrium volume, LA), LV systolic function (fractional shortening, FS%), left ventricle diastolic function (early diastolic filling wave, E; late diastolic filling wave, A; and E/A ratios), left ventricular mass (left ventricular mass indexed to height, LVMI), and geometry (relative wall thickness, RWT) were recorded. Fractional shortening (FS%), calculated as (LVEDD-LVESD)/LVEDD × 100 using parameters derived from M-Mode, was considered normal when ≥35%. The ejection fraction (EF) was calculated using the formula EF = (EDV–ESV)/EDV, where EDV (end-diastole volume) and ESV (end-systole volume) estimates were derived from the modified Simpson’s rule using the biplane method of disk. RWT was calculated as 2 × PWD/LVEDD [26] and considered increased for RWT > 0.42. LV mass was considered increased (LVH) based on age- and sex-specific 95th percentile values of LVMI (g/m^2.7^) (pediatric criteria) proposed by Khoury et al. [27] and Chinali et al. when LVMI is greater than 45 g/m^2.16^ (pediatric criteria) [28]. LV geometry was classified into four groups based on LV mass (normal or hypertrophied) and RWT (normal or increased) as follows: (1) normal geometry with normal LV mass and RWT; (2) remodeling with normal LV mass and increased RWT; (3) eccentric LVH with increased LV mass and normal RWT; (4) concentric LVH with both LV mass and RWT increased [24,29]. LV systolic function was considered decreased when LV ejection fraction (EF) was <53%. Diastolic dysfunction was classified according to ASE/EACVI 2016 Guidelines and Standards [25]. Cardiological evaluations, including complete echocardiographic assessments, were conducted at baseline (T0), after 1 year (T1), and after 2 years (T2). This study received approval from the local Ethics Committee (Comitato Etico Area Vasta Emilia Centro—AVEC, Bologna, Italy) (No. 207/2020/Oss/AOUBo, approved on 25 March 2020). Written informed consent was obtained from parents, and the methodology in this study adhered to relevant guidelines and regulations.

## 3. Statistical Analysis

Descriptive statistics were employed to characterize the patient cohort. Categorical variables are presented as numbers and relative percentages, while continuous variables are reported as means and standard deviations (SD) for normally distributed data or as medians and interquartile ranges (IQRs) for non-normally distributed data. Normality was assessed using the Kolmogorov–Smirnov test. Differences between categorical variables in the group data were analyzed using the chi-square test, and continuous variables were compared using either Student’s *t*-test or Mann–Whitney test, as appropriate. Linear regression models were utilized to model relationships between continuous variables. Statistical significance was defined at *p* < 0.05. Data analysis was performed using IBM Corp. Released 2016. IBM SPSS Statistics for Windows, Version 24.0, Armonk, NY: IBM Corp., and GraphPad Prism version 8.0.0 for Windows, San Diego, CA, USA.

## 4. Results

Ninety-two patients (50 boys, 54.3%; mean age: 11.4 ± 3 years) were enrolled. Table 1 displays the baseline characteristics of the patients. Most of the patients classified as Endocrine Disorders were followed for obesity or overweight in the absence of any other primary endocrine pathology.

**Figure 1:**
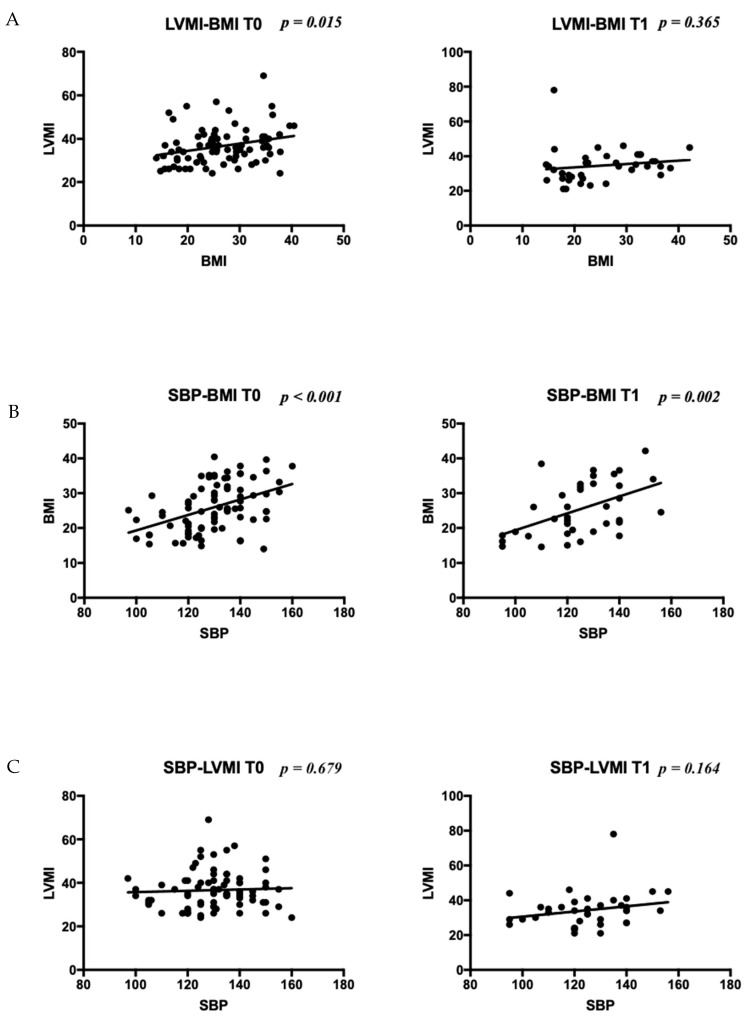
Correlation between BMI and LV mass (**A**), BMI and SBP (**B**), and SBP and LV mass (**C**) at T0 and T1. Legend: LVMI stands for left ventricular mass index; SBP stands for systolic blood pressure.

**Figure 2:**
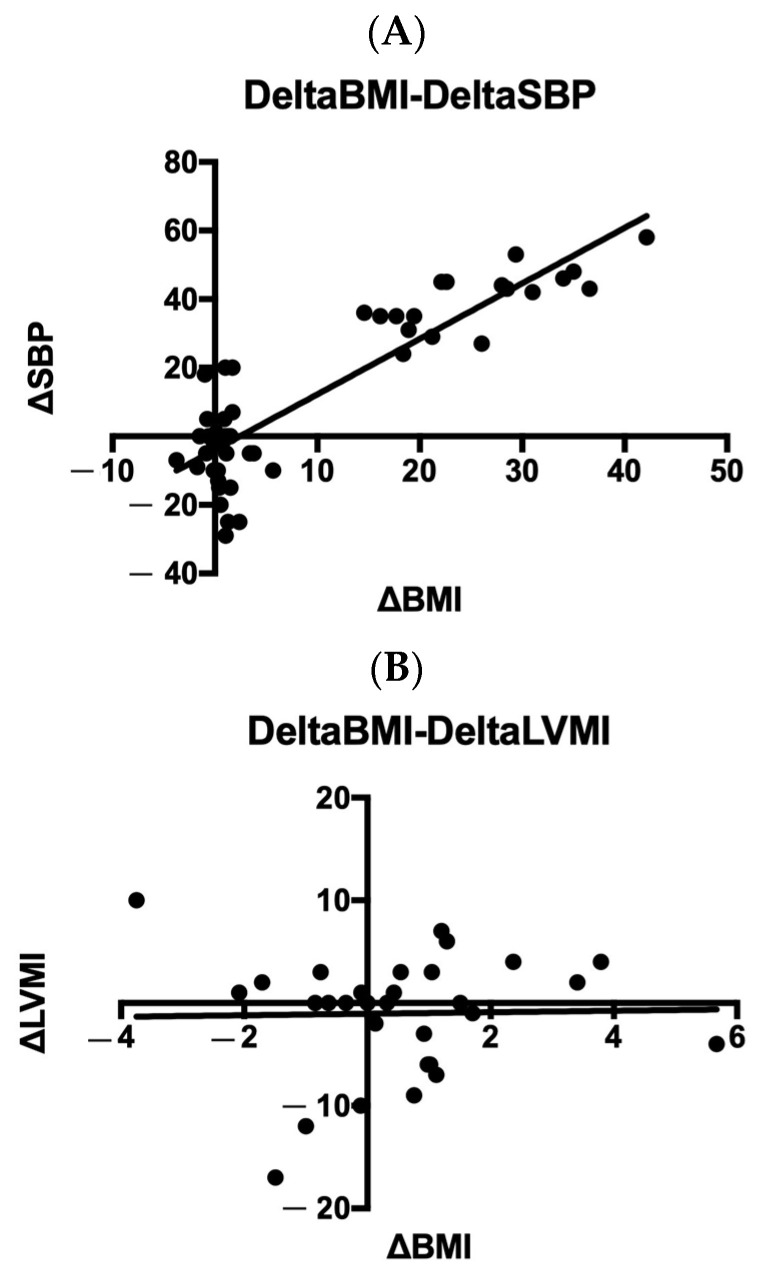
(**A**) Correlation between BMI and SBP variation at first follow-up; (**B**) correlation between BMI and LVMI variation at first follow-up. LVMI stands for LV mass index; SBP stands for systolic blood pressure.

**Figure 3:**
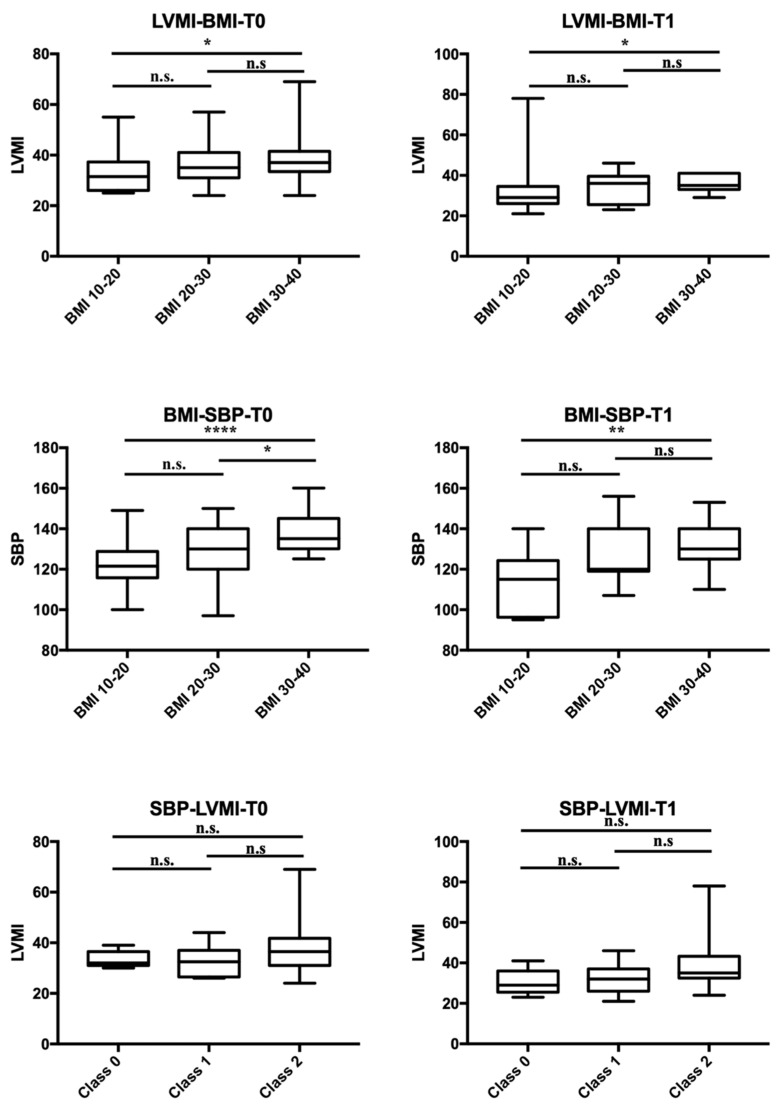
Comparison of LVMI (top panels) and SBP (middle panels) among groups based on BMI at T0 and T1, and LVMI and SBP at T0 and T1 (bottom panels) when patients were divided among level of SBP values (normal, high-normal, hypertension). LVMI stands for LV mass index; Class 0 stands for normotensive patients; Class 1 stands for patients with high-normal blood pressure; Class 2 stands for hypertensive patients; * = *p* ≤ 0.05; ** = *p* ≤ 0.01; **** = *p* ≤ 0.0001. n.s.: not significant.

Only a few of them had a different diagnosis of primary endocrine pathology: three type 1 diabetes, two polycystic ovary syndrome (PCOS), and two early puberty. Remarkably, blood pressure (BP) increased across BMI-related groups. Obese patients showed a higher prevalence of hypertension (HTN), with one patient having normal BP. In contrast, the distribution of BP categories was similar in the chronic kidney disease (CKD) and normal-weight groups. Given that endocrine and kidney diseases were the most frequent conditions, a comparison was made between the two groups (Table 2). At T0, patients with endocrine disorders (ED) exhibited significantly higher systolic blood pressure (SBP) (*p* < 0.001), a higher frequency of HTN (*p* < 0.001), higher BMI (*p* < 0.001), and a greater rate of obesity (*p* < 0.001) compared to CKD patients. At T1, absolute values of SBP and BMI remained higher in the ED group. Additionally, increased left ventricular mass index (LVMI) according to Chinali et al. was more prevalent in the ED patients at T1. Comparing patients with normal BP, high-normal BP, and HTN, BMI differed significantly (*p* < 0.01), particularly between patients with normal BP and those with HTN (*p* < 0.01, CI 2.75–9.15), while birth weight, gestational age, cardiac dimensions, LVMI and RWT did not significantly differ. No significant correlation was found between SBP and DBP and perinatal factors (birth weight, birth weight >10 centile and >90 centile, term/preterm/post term birth, delivery complications, twins, and neonatal adaptation), familial history of CVD, HTN, and CKD. However, positive correlations were found with BMI (*p* < 0.001, OR 1.18, CI 1.08–1.28), BMI ≥ 75th centile (*p* = 0.049, OR 2.8, CI 1.1–7.6), and BMI ≥ 95th centile (*p* < 0.001, OR 8.4, CI 2.3–28). Personal and familial risk factors for HTN are presented in the Supplementary Materials, Table S1. Cardiac geometry was altered in 2/10 (20%) of normotensive patients, 3/12 (25%) with high-normal BP, and 32/70 (46%) with HTN. Dividing the cohort into normal weight vs. overweight/obese (Table 3), normal geometry was significantly more frequent in normal-weight patients (*p* = 0.026). A significant positive correlation was found between BMI and LV mass at T0 (*p* = 0.015) and BMI and SBP at T0 and T1 (*p* < 0.001 and *p* = 0.002, respectively; Figure 1). Notably, a positive correlation was found between BMI and LVMI indexed by 2.7 and 2.16 (*p* = 0.012 and *p* = 0.002, respectively), but not with RWT nor fractional shortening (FS). No significant correlation was found between SBP and DBP, and LV hypertrophy (LVH) according to Khoury’s and Chinali’s definitions, pathological RWT, mean LVMI indexed by h^2.7^ and h^2.16^, RWT, and FS. A multivariate regression model adjusted for gender, BMI, SBP, and DBP revealed that only BMI was statistically significant in predicting LVMI^2.7^ and LVMI^2.16^ (*p* = 0.006 and *p* = 0.001). An amount of 45 out of 92 (48.9%) were evaluated at T1 (mean time 13 ± 4 months after T0), and 15/92 (16.3%) at T2 (mean time 14 ± 5 months after T1). During follow-up, the variation of BMI positively correlated with the variation of SBP, while not with the variation of LVMI (Figure 2). The evolution of cardiac mass and geometry is presented in Table S2. Dividing the patients into groups according to BMI (BMI 10–20, BMI 20–30, BMI 30–40), LVMI and SBP showed a positive correlation with BMI at T0 and T1, particularly between patients with BMI 10–20 and those with BMI 30–40. On the contrary, no significant correlation between LVMI and SBP was observed when patients were divided based on SBP values (normal, high-normal, hypertension) (Figure 3). DBP was not significantly correlated with BMI nor LVMI.

## 5. Discussion

The global burden of childhood hypertension has been on the rise over the past two decades, paralleling the increased prevalence of higher adiposity, reaching a peak of 7.9% in children aged 14 years [14]. Presently, hypertension and overweight collectively pose substantial public health challenges worldwide. Our study demonstrates a robust association between overweight, obesity, and elevated blood pressure (BP), with the severity escalating alongside BP values. Notably, obese subjects exhibit the highest prevalence of hypertension. BMI emerges as the most influential factor linked to cardiac organ damage in patients monitored for hypertension, while hypertension appears to have no significant impact on cardiac geometry or function. Consistent with this, systolic pressure is notably higher in patients with endocrine diseases compared to those with kidney diseases during longitudinal follow-up, as is the BMI. This underscores the importance of addressing the overweight and obesity in patients already at increased risk for cardiovascular disease [30]. Except for some patients diagnosed with DM1, PCOS, and early puberty, most of the patients classified as ED were being monitored for obesity or overweight in the absence of any other primary endocrine pathology. Specifically, none of them were found to have hyperthyroidism or hypercortisolism, two conditions that, due to hormonal imbalance, can directly contribute to arterial hypertension. Interestingly, the BP pattern in patients with nephropathy mirrors those with normal BMI. This might be attributed to the better compliance of these patients to medical treatment, including a low-salt diet and weight control, despite having a chronic pathology. While we did not find a significant correlation between hypertension and cardiac geometry or function, 39.8% of cases exhibited abnormal cardiac geometry, particularly in hypertensive patients. However, our findings align with prior studies reporting left ventricular hypertrophy (LVH) in 32.4–46.5% of hypertensive children [31]. In addition, Litwin et al. showed a linear correlation between waist-to-height ratio and cardiac mass [32,33]. Furthermore, our data suggest that greater LVMI and LVH according to Chinali’s definition persist more frequently in patients with increased BMI during longitudinal follow-up. The association between adiposity and cardiac abnormalities is supported by the significantly higher prevalence of abnormal cardiac geometry in overweight and obese patients. Crucially, in a multiple regression model including gender, BMI, SBP, and DBP, only excess weight significantly predicted left ventricular mass. As expected, altered geometry and higher left ventricular mass are the most common abnormalities in our population. In contrast, systolic and diastolic dysfunction were rare and unaffected by BMI. Although cardiac dysfunction was not highlighted in our study due to the short observational period, left ventricular altered geometry and hypertrophy are identified as the leading causes and initial steps towards long-term myocardial organ damage. This process begins as an adaptive response to increased left ventricular post-charge. Initially involving compensatory hemodynamic mechanisms, the underlying pathophysiological responses eventually lead to apoptosis of cardiomyocytes, resulting in reduced myocardial contractility and cardiac workload. The subsequent development of fibrosis, caused by fibroblast proliferation in the myocardial interstitium and perivascular region, predisposes individuals to the development of diastolic and systolic dysfunction of the left ventricle [1].

However, we cannot disregard the potential contribution of increased metabolic demand in this specific population. By increasing the sample size and including overweight and obese patients with and without systemic hypertension, we can obtain further insights and knowledge on this matter. Notably, diastolic pressure did not appear to impact cardiac injury, contrary to previous findings [34]. In adults, overweight and obesity can independently affect the heart irrespective of blood pressure levels. In these conditions, total and central blood volume indeed seem to increase, leading to left ventricular remodeling and hypertrophy [35]. Factors such as insulin resistance, the renin–angiotensin–aldosterone system, systemic inflammation, and sympathetic overdrive contribute to obesity-associated left ventricular dysfunction and remodeling [36,37]. Our data suggest that similar mechanisms may operate in younger patients. Despite the fact that a nomogram-based prognostic model indicates early life factors, family history, weight status, and lifestyle factors as predictors of hypertension in youth, our population did not show significant links between personal history and familial history for CVD and hypertension or cardiac damage [38]. However, a significant correlation was documented between BMI and cardiac mass, with cardiac mass being higher in patients with higher BMI, and this difference persisting over time. The same correlation was observed between SBP and BMI at the two time points. Remarkably, the variation of BMI positively modified systolic blood pressure but not cardiac mass during follow-up. This suggests that mechanisms leading to increased blood pressure may respond more rapidly to changes in adiposity than ventricular structural changes. The reversibility of blood pressure values is documented by the second year of follow-up, while the regression of cardiac abnormalities may take longer. A longer follow-up could confirm this hypothesis. Our findings suggest that the variation of weight, rather than the variation of blood pressure, may modify the progression of hypertension-related cardiac damage, regardless of the primary cause of hypertension. Early, focused interventions on body weight management, encompassing lifestyle changes, improved dietary habits, and increased physical activity, could modify blood pressure and subsequently cardiac injury, reducing cardiovascular morbidity and mortality. A healthy lifestyle and balanced nutrition plan may also positively impact mental health, which has been shown to be linked with dietary intake and physical activity in obese children [39,40,41]. Early school-based interventions focusing on nutrition could potentially alter the natural history of obesity-related metabolic syndrome, including hypertension and cardiac damage [42]. Longitudinal studies are imperative to assess the long-term association between BMI variation and hypertension and cardiac damage.

Our study presents some limitations. The main limitation of this study is its observational nature, not allowing us to assess the causal association between adiposity, cardiac injury and BP. Being a monocentric study limits the sample size, especially when the whole cohort is divided into subgroups. Another limitation may be represented by the prevalence of the overweight/obese group in our cohort: it exceeds the prevalence of the other etiologies of hypertension, potentially affecting the BMI of the entire cohort.

The lack of data about physical activity, dietary intervention and nutrition represents another limit of our study.

## 6. Conclusions

Our data underscore the profound impact of weight excess on blood pressure (BP) values and cardiac damage in children and adolescents with hypertension (HTN), irrespective of the underlying cause of elevated BP. Notably, while variations in weight positively correlate with changes in systolic blood pressure, there appears to be no immediate impact on short-term cardiac remodeling. These results emphasize the critical importance of promoting a healthy lifestyle and appropriate dietary habits from the early stages of life to mitigate the prevalence of overweight and obesity in the pediatric population and, consequently, alleviate the potential cardiovascular morbidity in future adult generations.

To substantiate these findings and further explore the efficacy of interventions, long-term, high-quality school-based studies are warranted. These studies should focus on promoting nutritional education and physical activity, aiming to enhance both the cardiometabolic and mental health profiles of obese and overweight children.

## Figures and Tables

**Table 1:** Baseline characteristics of patients.

Patients, n (boys n, %)	92 (50, 54.3%)			
Mean age (years) ± SD	11.4 ± 3			
Etiology, n (%)				
Overweight/obesity	51 (55)			
CKD	27 (29.3)			
OSAS/snoring	9 (9.8)			
Primary hypertension	5 (5.4)			
BMI, n (%)				
Normal weight	37 (40.2)			
Overweight	10 (10.9)			
Obese	45 (48.9)			
BP classification, n (%)				
Normal	10 (10.9)			
High-normal	12 (13)			
Hypertension	70 (76.1)			
BP classification according to diagnosis, n (%)	CKD	ED with normal weight	ED with overweight	ED obeses
Normal	3 (17.6)	3 (15)	0	1 (2.6)
High-normal	7 (41.2)	8 (40)	1 (25)	3 (7.9)
Hypertension	7 (41.2)	9 (45)	3 (75)	34 (89.5)
Cardiac morphology, n (%)				
Normal	57 (61.9)			
Remodeling	9 (9.8)			
Eccentric hypertrophy	17 (18.5)			
Concentric hypertrophy	9 (9.8)			
Ventricular function, n (%)				
Normal	74 (80.4)			
Systolic dysfunction	12 (13)			
Diastolic dysfunction	3 (3.3)			
Impaired relaxation	3 (3.3)			

Legend: CKD stands for chronic kidney disease; ED stands for endocrine disorders; BP stands for blood pressure; n stands for number of patients.

**Table 2:** BP and BMI data, and echocardiographic findings at the time of enrollment (T0) and first follow-up (T1) in patients divided into groups according to their diagnosis, with chronic kidney disease (CKD) and endocrine disorders (ED).

	CKD T0	ED T0	*p* Value T0	CKD T1	ED T1	*p* Value T1
SBP (mmHg), mean ± SD	119.68 ± 16.63	133.11 ± 12.29	<0.001 ^a^	113.5 ± 15.99	130.06 ± 14.12	0.010 ^a^
DBP (mmHg), mean ± SD	69.5 ± 11.2	72.4 ± 9.8	n.s	65.2 ± 10.8	72 ± 10.5	0.038 ^a^
BP categories, n (%)						
(1) Normal BP	3/17 (17.6%)	1/44 (2.3%)	0.029 ^b^	4/10 (40%)	3/15 (20%)	n.s.
(2) High Normal BP	7/17 (41.2%)	6/44 (13.6%)	0.018 ^b^	4/10 (40%)	6/15 (40%)	n.s.
(3) HTN	7/17 (41.2%)	37/44 (84.1%)	<0.001 ^b^	2/10 (20%)	6/15 (40%)	n.s.
BMI, mean ± SD	19.53 ± 4.42	30.98 ± 5.23	<0.001 ^a^	19.26 ± 5.47	32.75 ± 4.53	<0.001 ^a^
BMI categories, n (%)						
(1) Normal weight	15/17(88.2%)	5/44 (11.4%)	<0.001 ^b^	9/10 (90%)	0	-
(2) Overweight	1/17 (5.9%)	3/44 (6.8%)	n.s.	0	0	-
(3) Obesity	1/17 (5.9%)	36/44 (81.8%)	<0.001 ^a^	1/10 (10%)	15/15 (100%)	-
Echocardiographic findings						
LVMI (g/h^2.7^), mean ± SD	38.35 ± 18.05	37.18 ± 10.94	n.s.	33.18 ± 16.06	36.70 ±4.66	n.s.
LVMI (g/h^2.16^), mean ± SD	40.24 ± 9.90	45.92 ± 11.69	n.s.	34.5 ±10.16	46.11 ± 5.97	<0.001 ^a^
RWT, mean ± SD	0.37 ± 0.04	0.39 ± 0.11	n.s.	0.36 ± 0.08	0.38 ± 0.04	n.s.
FS%, mean ± SD	39.35 ± 4.02	39.04 ± 4.38	n.s.	36.2 ± 3.49	38.63 ± 4.38	n.s.
LVH (Khoury et al. [27]), %	17.6%	33.3%	n.s.	9.1%	29.4%	n.s.
LVH (Chinali et al. [28]), %	35.3%	45.2%	n.s.	20%	52.9%	0.039 ^b^
RWT > 0.42, %	11.8%	21.4%	n.s.	18.2%	17.6%	n.s.
FS% < 35%, %	10%	14%	n.s.	18.2%	3/17 (17.6%)	n.s.
E/A < 1,%	5%	4.7%	n.s.	9.1%	0	-
Normal LV geometry, %	76.4%	55.9%	n.s.	72.7%	58.8%	n.s.
Concentric hypertrophy, %	0%	11.6%	-	9.1%	5.9%	n.s.
Eccentric hypertrophy, %	11.8%	20.9%	n.s.	0	23.5%	-
Concentric remodeling, %	11.8%	11.6%	n.s.	18.2%	11.8%	n.s.

^a^ Mann–Whitney U Test; ^b^ Chi-square test; Legend: CKD stands for chronic kidney disease; ED stands for endocrine disorders; SBP stands for systolic blood pressure; DBP stands for diastolic blood pressure; BP stands for blood pressure; LVMI stands for left ventricular mass index; LVH stands for left ventricular hypertrophy; RWT stands for relative wall thickness; FS stands for fractional shortening; n.s. stands for not significant; n stands for number of patients.

**Table 3:** Echocardiographic findings in patients based on their BMI (normal weight vs. overweight/obese patients).

Parameters at T0	Normal Weight	Overweight/Obese	*p* Value
LVMI (g/h^2.7^), mean ± SD	38.4 ± 17.6	38.9 ± 9.6	n.s.
LVMI (g/h^2.16^), mean ± SD	41.3 ± 11.1	46.7 ± 11.4	n.s.
RWT, mean ± SD	0.37 ± 0.03	0.38 ± 0.08	n.s.
FS%, mean ± SD	38.1 ± 4.5	39.3 ± 4.3	n.s.
LVH (Khoury et al. [27]), n (%)	4/21 (19.1%)	16/46 (34.8%)	n.s.
LVH (Chinali et al. [28]), n (%)	6/21 (28.6%)	22/46 (47.8%)	n.s.
RWT > 0.42, n (%)	3/21 (14.3%)	10/46 (21.7%)	n.s.
FS% < 35%, n (%)	5/21 (23.8%)	5/46 (10.9%)	n.s.
E/A < 1, n (%)	1/21 (4.8%)	2/46 (4.3%)	n.s.
Normal LV geometry, n (%)	16/21 (76.2%)	24/46 (52.2%)	0.026 ^a^
Concentric hypertrophy, n (%)	2/21 (9.5%)	5/46 (10.8%)	n.s.
Eccentric hypertrophy, n (%)	2/21 (9.5%)	11/46 (23.9%)	n.s.
Concentric remodeling, n (%)	1/21 (4.8%)	6/46 (13.1%)	n.s.

^a^ Chi-square test; Legend: LVMI stands for left ventricular mass index; LVH stands for left ventricular hypertrophy; RWT stands for relative wall thickness; FS stands for shortening fraction; n.s. stands for not significant; n stands for number of patients.

## Data Availability

Data are contained within the article and Supplementary Materials.

## Supplementary Materials

The following supporting information can be downloaded at: https://www.mdpi.com/article/10.3390/nu15245079/s1, Table S1: Personal and familial risk factors for HTN. Table S2: Cardiac mass and geometry at first evaluation (T0), at first (T1) and second follow-up (T2).

## Abbreviations

HTN	hypertension
CV	cardiovascular
RF	risk factor
LV	left ventricular
LVH	left ventricular hypertrophy
BP	blood pressure
BMI	body mass index
CKD	chronic kidney disease
ED	endocrine disorders
OSAS	obstructive sleep apnea syndrome
CVD	cardiovascular diseases
SBP	systolic blood pressure
DBP	diastolic blood pressure
IVSD	interventricular septal width at end-diastole
LVEDD	left ventricle end-diastole diameter
LVESD	left ventricle end-systole diameter
PWD	posterior wall thickness at end-diastole
LA	left atrium volume
FS	fractional shortening
LVMI	left ventricular mass index
RWT	relative wall thickness
EF	ejection fraction
EDV	end-diastole volume
ESV	end-systole volume
SD	standard deviations
T0	first evaluation at enrollment time
T1	first follow-up
T2	second follow-up
SGA	small for gestational age
LGA	large for gestational age
