# Supplementary material for: Body Mass Index (BMI) Is the Strongest Predictor of Systemic Hypertension and Cardiac Mass in a Cohort of Children
Source: Nutrients. 2023 Dec 12;15(24):5079. doi: 10.3390/nu15245079 (PMC10745364; doi:10.3390/nu15245079)
Supplement: Supplementary file 1 [file nutrients-15-05079-s001.zip › nutrients-2695592-supplementary.pdf]

Supplementary Materials

Table S1: Personal and familial risk factors for HTN

**Perinatal History**	
**Birth Weight**	**Mean ± SD**
grams	3316 ± 717.6
**Percentile BW**	**Number of patients (%)**
<10° (SGA)	13/92 (14.1%)
10°- 90° (AGA)	52/92 (56.5%)
>90° (LGA)	27/92 (29.4%)
**Gestational Age**	**Mean ± SD**
Weeks	37.6 ± 2.6
**Classification by Gestional Age**	**Number of patients (%)**
Term Pregnancy	79/92 (85.9%)
Pre-term Pregnancy	13/92 (14.1%)
Post-term Pregnancy	0
**Twins** (n=92)	**Number of patients (%)**
Yes	6/92 (6.5%)
No	86/92 (93.5%)
**Delivery complications** (n=92)	**Number of patients (%)**
None	82/92 (89.2%)
Eclampsia	8/92 (8.6%)
Placenta previa	1/92 (1.1%)
Oligohydramnios	1/92 (1.1%)
**Neonatal Adaptation** (n= 92)	**Number of patients (%)**
Normal	74/92 (80.4%)
Need for Hospitalization	18/92 (19.6%)
Family History	

**First degree relatives with HTN** (n=92)	**Number of patients (%)**
Yes	42/92 (45.7%)
No	50/92 (54.3%)
**Second degree relatives with HTN** (n=92)	**Number of patients (%)**
Yes	34/92 (37%)
No	58/92 (63%)
**First degree relatives with diabetes mellitus** (n=92)	**Number of patients (%)**
Yes	11/92 (11.9%)
No	81/92 (88.1%)
**Second degree relatives with at least one among diabetes mellitus, dyslipidemia, obesity, CV diseases, kidney diseases** (n=92)	**Number of patients (%)**
Yes	32/92 (34.8%)
No	60/92 (65.2%)

Legends: SGA stands for small for gestational age; AGA stands for adequate for gestational age; LGA stands for large for gestational age.

Table S2: Cardiac mass and geometry at first evaluation (T0), at first (T1) and second follow-up (T2)

**Parameters**	**T0**	**T 1**	**T2**
**Number of patients**	92	45	14
**LVMI ($g/m^{2.7}$),** mean ± SD	37.4±12.3	34.5±9.5	36.1±13.7
**LVMI ($g/m^{2.16}$),** mean ± SD	43.9±11.2	40.4± 8.9	40.8±11.4
**RWT,** mean ± SD	0.38±0.11	0.39±0.06	0.37±0.12
**LVH ($g/m^{2.7}$)**, n (%)	27/92 (29.8%)	8/42 (19%)	4/14 (28.7%)
**LVH ($g/m^{2.16}$)**, n(%)	38/92 (41.8%)	12/40 (30%)	6/14 (42.8%)
**RWT >0.42**, n (%)	18/92 (19.4%)	11/45 (24.4%)	2/14 (14.3%)
**Normal geometry**, n (%)	55/92 (59.7%)	29/45 (64.4%)	8/14 (57.1%)
**Concentric hypertrophy**, n (%)	8/92 (9%)	5/45 (11.1%)	2/14 (14.3%)
**Eccentric hypertrophy**, n (%)	16/92 (17.9%)	4/45 (8.9%)	2/14 (14.3%)
**Concentric remodelling**, n (%)	7/92 (10.5%)	7/45 (15.6%)	1/14 (7.1%)

Legend: LVMI stands for left ventricular mass index; LVH stands for left ventricular hypertrophy; RWT stands for relative wall thickness; FS stands for shortening fraction; n stands for number of patients.
